# Incidence, Characteristics and Survival Rates of Bladder Cancer after Rectosigmoid Cancer Radiation

**DOI:** 10.3390/cancers16132404

**Published:** 2024-06-29

**Authors:** Mario de Angelis, Carolin Siech, Francesco Di Bello, Natali Rodriguez Peñaranda, Jordan A. Goyal, Zhe Tian, Nicola Longo, Felix K. H. Chun, Stefano Puliatti, Fred Saad, Shahrokh F. Shariat, Mattia Longoni, Giorgio Gandaglia, Marco Moschini, Francesco Montorsi, Alberto Briganti, Pierre I. Karakiewicz

**Affiliations:** 1 Cancer Prognostics and Health Outcomes Unit, Division of Urology, University of Montréal Health Center, Montréal, QC H2X 3E4, Canada; deangelis.mario@hsr.it (M.d.A.); carolin.siech@kgu.de (C.S.); fran.dibello12@gmail.com (F.D.B.); natalirodriguez647@gmail.com (N.R.P.); jordan.goyal@umontreal.ca (J.A.G.); zhe.tian@umontreal.ca (Z.T.); fred.saad@umontreal.ca (F.S.); 2 Division of Experimental Oncology, Unit of Urology, URI, IRCCS Ospedale San Raffaele, 20132 Milan, Italy; longoni.mattia@hsr.it (M.L.); gandaglia.giorgio@hsr.it (G.G.); moschini.marco@hsr.it (M.M.); montorsi-francesco@hsr.it (F.M.); briganti.alberto@hsr.it (A.B.); 3 Vita-Salute San Raffaele University, 20132 Milan, Italy; 4 Department of Urology, University Hospital, Goethe University Frankfurt, 60590 Frankfurt am Main, Germany; felix.chun@kgu.de; 5 Department of Neurosciences, Science of Reproduction and Odontostomatology, University of Naples Federico II, 80131 Naples, Italy; nicola.longo@unina.it; 6 Department of Urology, Ospedale Policlinico e Nuovo Ospedale Civile S. Agostino Estense Modena, University of Modena and Reggio Emilia, 41121 Modena, Italy; stefano.puliatti@unimore.it; 7 Department of Urology, Comprehensive Cancer Center, Medical University of Vienna, 1090 Vienna, Austria; shahrokh.shariat@meduniwien.ac.at; 8 Department of Urology, Weill Cornell Medical College, New York, NY 10065, USA; 9 Department of Urology, University of Texas Southwestern Medical Center, Dallas, TX 75390, USA; 10 Hourani Center of Applied Scientific Research, Al-Ahliyya Amman University, Amman 19328, Jordan

**Keywords:** bladder cancer, rectal cancer, external beam radiation therapy, radiotherapy

## Abstract

**Simple Summary:**

External beam radiation therapy with or without systemic chemotherapy is a well-established treatment modality in patients with non-metastatic localized or locally advanced rectosigmoid cancer, with improved cancer control and survival rates. However, it has been previously extensively demonstrated that there is an association between radiation exposure and the risk of radiation-induced malignances. Specifically, previous studies demonstrated the increased risk of bladder cancer after radiation therapy for rectal cancer relative to radiation-unexposed counterparts. However, these studies relied on historical cohorts and also relied on more historical radiation delivery techniques. Moreover, only one of these studies addressed bladder cancer-specific survival rates in patients with bladder cancer after rectal cancer according to previous radiation exposure for rectal cancer. The aim of this study was to evaluate the effect of radiation therapy for rectal cancer on risk of subsequent bladder cancer relative to radiation-unexposed counterparts, relying on the most contemporary cohort of patients. We ascertained that although historical radiation therapy for rectal cancer predisposed patients to higher subsequent bladder cancer rates, contemporary external beam radiation therapy for rectal cancer is not associated with increased subsequent bladder cancer risk. Moreover, when bladder cancer after rectal cancer occurs, bladder cancer-specific mortality rates are not affected by previous radiation exposure for rectal cancer.

**Abstract:**

Background: Historical external beam radiation therapy (EBRT) for rectosigmoid cancer (RCa) predisposed patients to an increased risk of secondary bladder cancer (BCa). However, no contemporary radiotherapy studies are available. We addressed this knowledge gap. Materials and methods: Within the Surveillance, Epidemiology, and End Results database (2000–2020), we identified non-metastatic RCa patients who either underwent radiotherapy (EBRT+) or did not (EBRT-). Cumulative incidence plots and multivariable competing risk regression models (CRR) were fitted to address rates of BCa after RCa. In the subgroup of BCa patients, the same methodology addressed BCa-specific mortality (BCSM) according to EBRT exposure status. Results: Of the 188,658 non-metastatic RCa patients, 54,562 (29%) were EBRT+ vs. 134,096 (73%) who were EBRT-. In the cumulative incidence plots, the ten-year BCa rates were 0.7% in EBRT+ vs. 0.7% in EBRT- patients (*p* = 0.8). In the CRR, EBRT+ status was unrelated to BCa rates (multivariable HR: 1.1, *p* = 0.8). In the subgroup of 1416 patients with BCa after RCa, 443 (31%) were EBRT+ vs. 973 (69%) who were EBRT-. In the cumulative incidence plots, the ten-year BCSM rates were 10.6% in EBRT+ vs. 12.1% in EBRT- patients (*p* = 0.7). In the CRR, EBRT+ status was unrelated to subsequent BCSM rates (multivariable HR: 0.9, *p* = 0.9). Conclusion: Although historical EBRT for RCa predisposed patients to higher BCa rates, contemporary EBRT for RCa is not associated with increased subsequent BCa risk. Moreover, in patients with BCa after RCa, exposure to EBRT does not affect BCSM.

## 1. Introduction

Colorectal cancer is the third most commonly diagnosed cancer in the Western world. Among these, tumors that develop in the rectum or rectosigmoid junction account for approximately one-third of all colorectal cancers. Despite being very similar to other sites in terms of histology, risk factors, and tumorigenesis, rectal cancer can be considered a separate entity due to its peculiar anatomic location, blood supply, and lymphatic drainage, which require different surgical and non-surgical management [1,2,3]. Surgery with curative intent is still considered the gold standard treatment for rectal adenocarcinoma [4]. However, current guidelines recommend external beam radiation therapy (EBRT) alone or in combination with systemic chemotherapy in non-metastatic localized and locally advanced rectosigmoid cancer patients [5,6]. Specifically, neoadjuvant radiotherapy is indicated in cases of locally advanced disease (cT3 or more), lymph node invasion, or where total mesorectal incision is difficult or not possible. Indeed, the role of neoadjuvant radiation therapy is to reduce tumor volume and allow for surgery with safe surgical margins and also better preservation of the anal sphincter, aiming to preserve continence. Administration of radiation therapy with or without systemic chemotherapy resulted in higher rates of pathological complete response and even improved survival rates [7,8]. Indeed, previous trials demonstrated the added value of neoadjuvant chemotherapy before surgery vs. surgery alone. For example, the Swedish Rectal Cancer Trial showed reduced rates of local recurrence and improved survival relative to rectal cancer patients who underwent surgery alone [7]. Similarly, the European Organization for Research and Treatment of Cancer (EORTC) trial recorded a highly statistically significant and clinically meaningful local recurrence-free survival in radiation-exposed patients [9]. Similar considerations were made by the Stockholm Colorectal Cancer Study Group, who investigated the effect of radiation therapy on cancer control rates in rectal cancer patients in two randomized control rials (Stockholm I [10,11] and Stockholm II [10,11]). Specifically, radiation therapy significantly reduced rectal carcinoma deaths in both trials and also improved overall survival in the Stockholm II trial. Regarding postoperative radiation therapy, the combination of radiotherapy plus systemic chemotherapy is associated with improved cancer control rates compared to surgery alone [12]. Preoperative treatments include short-course radiotherapy, short-course chemoradiotherapy, long-course radiotherapy, and long-course chemoradiotherapy. Among them, short-course preoperative radiotherapy consists of 25 Gy in five consecutive days, while long-course preoperative chemoradiotherapy consists of 50.4 Gy in five weeks and three days with concurrent chemotherapy, which has been applied most widely in recent years [13]. In a recent meta-analysis, short- and long-course preoperative treatments seemed comparable for the management of rectal cancer in terms of outcomes such as survival, recurrence, and complications [13]. Taking these observations into account, the utilization of radiation therapy in the management of rectosigmoid cancer has been extensively used in the last few decades [14,15]. On the other hand, adjuvant radiotherapy can be proposed in cases of local excision, a high risk of local recurrence, and positive surgical margins with advanced disease [5,16]. However, although radiotherapy significantly improved rectal cancer-specific survival, overall survival rates were not different due to an increase in other causes of death such as secondary malignancies after radiation therapy [8]. Indeed, it is of note that EBRT is known to predispose patients to a higher risk of secondary bladder cancer when EBRT is delivered in the pelvis [17,18,19,20,21,22,23,24,25]. Specifically, the only three studies that addressed this concept in the context of rectosigmoid cancer were historical in nature and relied on historical EBRT delivery techniques [23,24,25]. Among these, only one addressed survival rates in patients with bladder cancer diagnosis after rectosigmoid cancer treatment [25]. As a result, it is unknown to what extent contemporary EBRT for rectosigmoid cancer predisposes patients to subsequent bladder cancer incidences. Moreover, it is unknown whether bladder cancer after contemporary EBRT for rectosigmoid cancer is more aggressive than bladder cancer after rectosigmoid cancer without previous EBRT. We addressed these knowledge gaps and hypothesized that contemporary EBRT for rectosigmoid cancer may result in lower subsequent bladder cancer rates compared to rates of historical EBRT for rectosigmoid cancer that were recorded in previous studies. Moreover, we also postulated that bladder cancer in EBRT for rectosigmoid cancer patients is associated with similar survival rates relative to EBRT-unexposed counterparts. We relied on the Surveillance, Epidemiology, and End Results (SEER) database (2000–2020) to test our hypotheses.

## 2. Materials and Methods

### 2.1. Study Population

Within the SEER database (2000–2020), we identified patients ≥18 years diagnosed with rectosigmoid cancer adenocarcinoma (International Classification of Disease for Oncology [ICD-O] code 18.7, 19.9, 20.9; ICD-O-3 histology codes: 8140, 8210, 8260, 8261, 8262, 8263) as their first malignancy. Subsequently, all patients were stratified according to exposure or no exposure to EBRT for rectosigmoid cancer. Moreover, diagnoses of subsequent bladder cancer were recorded (ICD-O site code C67.0–C67.9, any histology). Patients with unknown age at diagnosis, unknown vital status, unknown rectal cancer stage at presentation, unknown systemic chemotherapy administration, radiotherapy utilization other than EBRT as well as all autopsy or death certificate-only cases were excluded.

### 2.2. Statistical Analyses

Cumulative incidence plots illustrated bladder cancer rates after rectosigmoid cancer according to EBRT-exposed vs. EBRT-unexposed status. Thereafter, we fitted multivariable competing risk regression models predicting bladder cancer incidence after rectosigmoid cancer according to the presence or absence of EBRT exposure. Standard multivariable adjustment relied on age at diagnosis, sex, rectosigmoid cancer stage at presentation and systemic chemotherapy exposure. Additional adjustments relied on other-cause mortality. Subsequently, subgroup analyses examined bladder cancer-specific mortality according to EBRT exposure status: ERBT-exposed vs. EBRT-unexposed. Here, death was defined according to the SEER mortality code as bladder cancer-specific mortality (death attributable to bladder cancer) or other-cause mortality (death attributable to any other cause). Cumulative incidence plots illustrated bladder cancer-specific mortality rates according to EBRT-exposed vs. EBRT-unexposed status. Subsequently, multivariable competing risk regression models predicting bladder cancer-specific mortality were fitted according to EBRT-exposed vs. EBRT-unexposed status. Standard multivariable adjustments relied on age at bladder cancer diagnosis, sex, bladder cancer stage at presentation, histological subtype and systemic chemotherapy exposure. An additional adjustment was made for other-cause mortality. All tests were two-sided, with a significance level of *p* < 0.05. R software environment for statistical computing and graphics (R version 4.2.2, R Foundation for Statical Computing, Vienna, Austria) was used for all analyses [26].

## 3. Results

### 3.1. Descriptive Characteristics of the Study Population

Overall, within the SEER database, we identified 188,658 non-metastatic rectosigmoid cancer patients between 2000 and 2020 (Table 1). Among these, 54,562 (29%) were EBRT-exposed vs. 134,096 (71%) who were EBRT-unexposed. Relative to the EBRT-unexposed patients, the EBRT-exposed patients with prior rectosigmoid cancer were younger (61 vs. 65 years) and a higher proportion harbored locally advanced stage disease (68% vs. 39%). Moreover, a higher proportion of EBRT-exposed patients were also exposed to systemic chemotherapy (93%) relative to their EBRT-unexposed counterparts (22%).

### 3.2. Cumulative Incidence Plots Addressing Bladder Cancer Rates after Rectosigmoid Cancer According to External Beam Radiation Therapy Exposure

**Figure 1:**
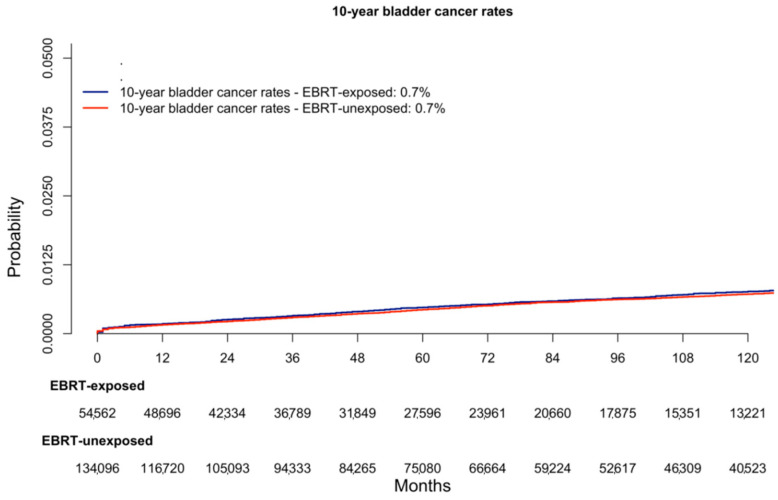
Cumulative incidence plot depicting ten-year bladder cancer rates in rectal cancer patients according to external beam radiation therapy exposure (exposed vs. unexposed).

In the cumulative incidence plots, ten-year bladder cancer rates of 0.7% in EBRT-exposed patients vs. 0.7% in EBRT-unexposed patients were recorded (*p* = 0.8, Figure 1). In the multivariable competing risk regression models, after accounting for other-cause mortality and all standard covariates, EBRT exposure status for rectosigmoid cancer was unrelated to subsequent bladder cancer diagnosis (Hazard Ratio: 1.1, 95% Confidence Interval: 0.8–1.3, *p*-value = 0.8, Table 2).

### 3.3. Subgroup Analyses Addressing Bladder Cancer after Rectosigmoid Cancer

#### 3.3.1. Baseline Characteristic of Rectosigmoid Cancer Patients with Subsequent Bladder Cancer Diagnosis

Between 2000 and 2020, we identified 1416 patients with bladder cancer after rectosigmoid cancer who either were EBRT-exposed or EBRT-unexposed due to rectosigmoid cancer (Table 3). Among these, 443 (31%) were EBRT-exposed vs. 973 (69%) who were EBRT-unexposed. Relative to the EBRT-unexposed patients, the EBRT-exposed patients with prior rectosigmoid cancer were younger (72 vs. 76 years) and a higher proportion harbored metastatic stage disease at presentation (5% vs. 3%). Moreover, a lower proportion of EBRT-exposed patients were also exposed to systemic chemotherapy (15%) relative to their EBRT-unexposed counterparts (21%). No statistically significant or clinically meaningful differences were recorded regarding histological subtype (*p* = 0.9) and surgical management (*p* = 0.7) between EBRT-exposed vs. EBRT-unexposed rectosigmoid cancer patients with subsequent bladder cancer diagnosis (Table 3).

#### 3.3.2. Survival Analyses in Bladder Cancer Patients with Previous Rectosigmoid Cancer According to Eternal Beam Radiation Therapy Exposure

**Figure 2:**
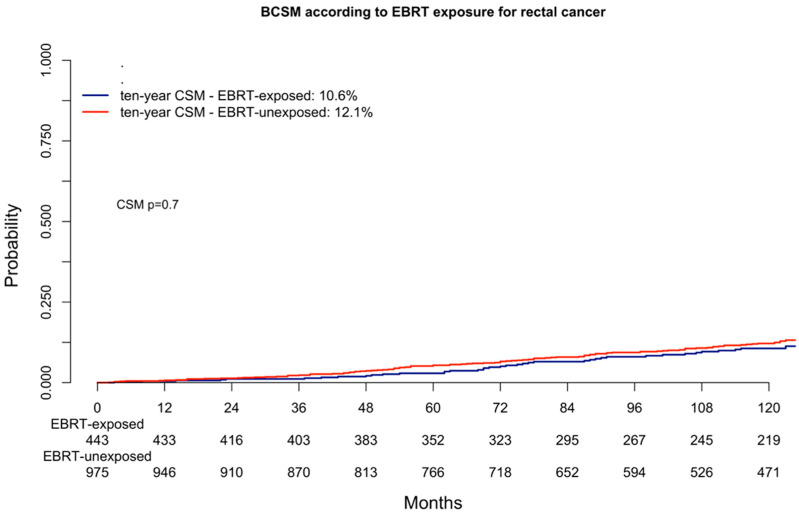
Cumulative incidence plot depicting bladder cancer-specific mortality (BCSM) in patients with bladder cancer diagnosis after rectal cancer according to external beam radiation therapy exposure (exposed vs. unexposed).

In the cumulative incidence plots, ten-year bladder cancer-specific mortality rates of 10.6% in EBRT-exposed patients vs. 12.6% in EBRT-unexposed patients were recorded (*p* = 0.7, Figure 2). In the multivariable competing risk regression models after accounting for other-cause mortality and all standard covariates, EBRT exposure status for rectosigmoid cancer was unrelated to higher bladder cancer-specific mortality rates (Hazard Ratio: 1.0, 95% Confidence Interval: 0.7–1.1, *p*-value = 0.9, Table 4).

## 4. Discussion

Historical studies demonstrated higher rates of bladder cancer after EBRT for rectosigmoid cancer [23,24,25,27]. In the current study, we tested for contemporary bladder cancer rates after rectosigmoid cancer according to EBRT exposure. Moreover, we also tested for bladder cancer-specific morality differences in patients with bladder cancer after previous rectosigmoid cancer according to EBRT exposure. We relied on the most contemporary SEER cohort (2000–2020) and made several noteworthy observations.

First, within the current sample of 188,658 rectosigmoid cancer patients, 54,562 (29%) were EBRT-exposed vs. 134,096 (71%) who were EBRT-unexposed. In historical reports, the proportion of EBRT-exposed rectosigmoid cancer patients ranged from 32 to 44% [23,24,25]. However, in these historical studies, radiotherapy definition included both EBRT and brachytherapy [23,24]. As a result, the lack of homogeneity in radiation treatment among rectal cancer patients will inevitably lead to more diluted and weaker evidence. Conversely, only one of these studies relied on the same radiotherapy definition that was used in the current study, namely EBRT alone. Specifically, Guan et al. recorded a similar proportion of EBRT-exposed patients (32%) as in the current study (29%) [25]. Moreover, the current data indicate that EBRT-exposed patients were younger (median age 61 vs. 65) and a higher proportion harbored locally advanced rectosigmoid cancer. These observations are consistent with previous reports, where median age in EBRT-exposed patients for rectosigmoid cancer ranged between 60 and 66 years and the proportions of patients with locally advanced rectosigmoid cancer ranged from 46 to 71% [23,24,25,28]. The recorded rates of EBRT utilization for rectosigmoid cancer in the current study as well as in previous studies are consistent with international guideline recommendations that support EBRT alone or in combination with systemic therapy predominantly in patients with locally advanced stage disease [6]. Ongoing recommendations of EBRT in contemporary patients with rectosigmoid cancer along with the unknown risk of subsequent bladder cancer in such settings validate the clinical relevance of the current study.

Second, we addressed rates of bladder cancer according to EBRT-exposed vs. EBRT-unexposed status in patients with prior rectosigmoid cancer. Here, we observed equally marginal bladder cancer rates between the two groups (0.7% in both groups). Based on the rarity of bladder cancer after rectosigmoid cancer according to presence or absence of EBRT, only large-scale population-based analyses such as those relying on the SEER database or the National Cancer Database can provide sufficient numbers of observations and events. After most complete multivariable adjustments, as well as adjustments for other-cause mortality, we recorded that EBRT exposure for rectosigmoid cancer was unrelated to subsequent bladder cancer diagnosis (multivariable Hazard Ratio: 1.1, *p*-value = 0.9). This observation is not consistent with previous studies, where an association between EBRT exposure for rectal cancer and subsequent bladder cancer diagnosis was observed. Specifically, relative to EBRT-unexposed patients, Guan et al. recorded 1.7-fold higher rates of bladder cancer in EBRT-exposed patients with prior rectosigmoid cancer [25]. Similarly, within their study, Li et al. reported 1.5-fold higher rates of bladder cancer rates in EBRT-exposed patients with prior rectosigmoid cancer relative to EBRT-unexposed counterparts [24]. Finally, Warschkow et al. also found a strong association between EBRT exposure status and bladder cancer after rectosigmoid cancer (multivariable Hazard Ratio: 1.5) [23]. As a result, it may be postulated that contemporary EBRT protocols for rectosigmoid cancer are safer than historical protocols. Based on the absence of recorded differences in bladder cancer rates after rectosigmoid cancer according to EBRT exposure, it appears that EBRT-exposed patients do not require specific regimens to monitor for potential subsequent bladder cancer diagnosis. To the best of our knowledge, the current study provides the most contemporary and most robust data supporting this recommendation.

Third, additional interesting observations were made regarding clinical characteristics in bladder cancer patients according to previous EBRT exposure. Specifically, relative to EBRT-unexposed patients, a higher proportion of EBRT-exposed patients harbored bladder cancer of a metastatic stage at presentation (5 vs. 2%). This observation is consistent with previous reports, where bladder cancer diagnosis after previous EBRT exposure is associated with a more advanced stage at presentation relative to EBRT-unexposed counterparts [21,22,29,30,31,32]. Indeed, it has been previously demonstrated in vitro that radiation exposure is associated with higher rates of p53 mutations and, consequently, also higher rates of high-grade bladder cancer [33,34,35]. Additionally, not only were the rates of bladder cancer after EBRT for rectosigmoid cancer not different between EBRT-exposed and EBRT-unexposed patients, but also, the histological subtype of bladder cancer (namely urothelial vs. non-urothelial) in both EBRT-exposed vs. EBRT-unexposed patients was also not different. Specifically, in both EBRT-exposed and EBRT-unexposed patients, the vast majority harbored the urothelial subtype (93%). Conversely, the proportions of non-urothelial bladder cancer histological subtypes were equally lower (7%). This observation is also of relevant interest, since it suggests that previous EBRT exposure is not associated with an increased risk of non-urothelial histological subtypes with more aggressive behavior. To the best of our knowledge, this is the first study addressing pathological characteristics in bladder cancer patients with a previous diagnosis of rectosigmoid cancer according to EBRT exposure status. As a result, a direct comparison with previous studies cannot be made, since they do not exist.

Fourth, in the final part of the current study, we tested for bladder cancer-specific mortality differences according to EBRT exposure. Specifically, the rates of bladder cancer-specific mortality according to EBRT-exposed vs. EBRT-unexposed status were, respectively, 10.6% vs. 12.1% (*p* = 0.7). After most complete multivariable adjustments, EBRT exposure was unrelated to bladder cancer-specific mortality (multivariable Hazard Ratio: 1.0, *p*-value = 0.9). These observations indicate that when bladder cancer is diagnosed after previous rectosigmoid cancer, bladder cancer-specific mortality is unrelated to EBRT exposure. To the best of our knowledge, only one other study tested survival rates in patients who developed bladder cancer after rectosigmoid cancer according to previous EBRT exposure. Specifically, Guan et al. relied on more historical data from the SEER database (1975–2013), where bladder cancer after previous rectosigmoid cancer was also unrelated to EBRT exposure (*p* = 0.2) [25]. However, in that historical report, no multivariable adjustment was made for a variety of important confounding variables that were accounted for in the current study. Moreover, in the previous study, an adjustment for other-cause mortality was not made. Conversely, relative to that historical study, the advantage of the current study relies in its methodology of competing risk regression, where not only is a standard multivariable adjustment made, but bladder cancer-specific mortality rates are also adjusted for other-cause mortality. Therefore, our findings provide the most robust data describing the natural history of subsequent bladder cancer after rectosigmoid cancer according to EBRT status. As a result, the combination of the historical study’s data and the current study’s data provides stronger evidence attesting to no survival differences in secondary bladder cancer according to EBRT exposure for rectosigmoid cancer.

Taken together, the current analyses provide very important take-home messages regarding bladder cancer after previous rectosigmoid cancer according to EBRT exposure. First, bladder cancer after previous rectosigmoid cancer represents an extremely rare entity as evidenced by the rate of 0.7% in ten years. Additionally, bladder cancer rates after rectosigmoid cancer are not influenced by previous EBRT exposure. These observations are of great interest since they indicate that contemporary EBRT protocols are clearly safer than historic EBRT when bladder cancer after rectosigmoid cancer represents the endpoint of interest. Consequently, our observations suggest that EBRT-exposed patients with prior rectosigmoid cancer do not require specific follow-up regimens to monitor for potential subsequent bladder cancer diagnosis. Finally, in the subgroup of patients who developed bladder cancer after previous rectosigmoid cancer, bladder cancer-specific survival rates are virtually the same regardless of radiation exposure.

Despite the novelty of our findings, several limitations need to be acknowledged. First, this study is retrospective in nature. However, this limitation is shared with all previous reports addressing the topic of EBRT-induced malignances within large population-based data repositories [17,18,19,20,21,22,23,24,25,27]. The second limitation consists of the lack of detail regarding type, dose and timing of EBRT. Additionally, only limited information about the type and extent of surgery as well as the type and duration of systemic therapy is available. Similarly, regarding secondary bladder cancer, only limited information about the type and extent of transurethral resection, the extent of lymphadenectomy at radical cystectomy as well as the type and duration of systemic therapy is available. All of the above limitations are the same as those in all previous population-based analyses that relied on the SEER database as well as the National Cancer Database [10,12,13,14,15,16,17,18,19,21]. Third, the SEER database does not provide specific information about baseline comorbidities. As a result, more detailed analyses, where comorbidities could be applied, were not possible. Finally, the SEER database does not provide any secondary cancer control endpoint, such as recurrence-free survival and metastasis-free survival.

## 5. Conclusions

Although historical EBRT for rectosigmoid cancer predisposed patients to higher bladder cancer rates, contemporary EBRT for rectosigmoid cancer is not associated with increased bladder cancer risk. Moreover, in patients with bladder cancer after rectosigmoid cancer, exposure to EBRT does not affect bladder cancer-specific mortality.

## Figures and Tables

**Table 1:** Baseline characteristics of 188,658 non-metastatic rectal cancer patients treated from Surveillance, Epidemiology, and End Results (SEER) database (2000–2020).

Characteristic	EBRT-Exposed 54,562 (29%) ^1^	EBRT-Unexposed 134,096 (71%) ^1^	*p*-Value ^2^
Age	61 (52, 70)	65 (55, 75)	<0.001
Gender			<0.001
Female	20,770 (38%)	60,706 (45%)	
Male	33,792 (62%)	73,390 (55%)	
Rectal cancer stage			
Localized	17,272 (32%)	81,317 (61%)	
Locally advanced	37,290 (68%)	52,779 (39%)	
Systemic chemotherapy			<0.001
Yes	50,750 (93%)	29,857 (22%)	
Race/ethnicity			<0.001
Caucasians	37,247 (68%)	91,631 (68%)	
Hispanics	7007 (13%)	15,238 (11%)	
African Americans	4435 (8%)	11,355 (9%)	
Asians	5261 (10%)	14,045 (10%)	
Others	612 (1%)	1827 (2%)	

^1^ Median (IQR); n (%). ^2^ Wilcoxon rank sum test; Pearson’s Chi-square test.

**Table 2:** Competing risk regression models testing risk of bladder cancer after rectal cancer in 188,658 patients according to previous EBERT exposure for rectal cancer and adjusted for overall mortality, in addition to standard multivariable adjustment for age at diagnosis, sex, rectal cancer stage and systemic chemotherapy exposure.

EBRT Exposure	HR	*p*-Value
EBRT exposed (vs. EBRT unexposed)	1.1 (0.8–1.3)	0.8

**Table 3:** Baseline characteristics of 1416 patients with bladder cancer diagnosis after rectal cancer between 2000 and 2020 from Surveillance, Epidemiology, and End Results (SEER) database.

Characteristic	EBRT-Exposed 443 (31%) ^1^	EBRT-Unexposed 973 (69%) ^1^	*p*-Value ^2^
Age	72 (65, 78)	76 (69, 81)	<0.001
Gender			0.7
Male	356 (80%)	790 (81%)	
Female	87 (20%)	183 (19%)	
Stage			0.04
Localized	367 (83%)	786 (81%)	
Regional	46 (10%)	137 (14%)	
Distant	23 (5%)	29 (3%)	
Unstaged	7 (2%)	21 (2%)	
Histological subtype			0.9
Urothelial	413 (93%)	905 (93%)	
Non-urothelial	30 (7%)	68 (7%)	
Systemic chemotherapy			0.02
Yes	68 (15%)	200 (21%)	
Surgical treatment			0.7
None	36 (8%)	77 (8%)	
Pelvic exenteration	3 (<1%)	3 (<1%)	
RC	21 (5%)	53 (5%)	
TURBT	383 (86%)	840 (86%)	

^1^ Median (IQR); n (%). ^2^ Wilcoxon rank sum test; Pearson’s Chi-square test; Fisher’s exact test.

**Table 4:** Competing risk regression models testing bladder cancer-specific mortality, adjusted for other-cause mortality in patients with bladder cancer diagnosis after rectal cancer divided according to previous EBRT exposure. Covariates consisted of age at bladder cancer diagnosis, bladder cancer stage, systemic chemotherapy administration for bladder cancer and histological subtype (urothelial vs. non-urothelial).

EBRT Exposure	HR	*p*-Value
EBRT exposed (vs. EBRT unexposed)	0.9 (0.7–1.1)	0.9

## Data Availability

The data presented in this study are available in this article.
